# Mitochondrial DNA sequencing and large-scale genotyping identifies *MT-ND4* gene mutation m.11696G>A associated with idiopathic oligoasthenospermia

**DOI:** 10.18632/oncotarget.17675

**Published:** 2017-05-08

**Authors:** Juan Ji, Miaofei Xu, Zhenyao Huang, Lei Li, Hongxiang Zheng, Shuping Yang, Shilin Li, Li Jin, Xiufeng Ling, Yankai Xia, Chuncheng Lu, Xinru Wang

**Affiliations:** ^1^ State Key Laboratory of Reproductive Medicine, Institute of Toxicology, Nanjing Medical University, Nanjing 210029, China; ^2^ Key Laboratory of Modern Toxicology of Ministry of Education, School of Public Health, Nanjing Medical University, Nanjing 210029, China; ^3^ Department of Children Health Care, Nanjing Maternity and Child Health Care Hospital Affiliated to Nanjing Medical University, Nanjing 210029, China; ^4^ State Key Laboratory of Genetic Engineering and Ministry of Education Key Laboratory of Contemporary Anthropology, School of Life Sciences, Fudan University, Shanghai 200433, China

**Keywords:** oligoasthenospermia, mitochondrial DNA, genetic variant, haplogroup

## Abstract

Genetic variants of mitochondrial DNA (mtDNA) were implicated to be associated with male infertility. Our previous whole mitochondrial genome sequencing and association study has identified two susceptibility mtDNA variants for oligoasthenospermia in Han Chinese men. In this study, we tested promising associations in an extended validation using 670 idiopathic oligoasthenospermia cases and 793 healthy controls to identify additional risk variants. We found that the genetic variant of m.11696G>A showed significantly higher frequency in the case group than that in the control group (odds ratio (OR) 2.21, 95% CI 1.21-4.04) (*P*=7.90×10^−3^). To elucidate the exact role of the genetic variants in spermatogenesis, two main sperm parameters (sperm count and motility) were taken into account. We found that m.11696G>A was associated with low sperm motility, with the OR of 2.38 (95 % CI 1.27-4.46) (*P* =5.22×10^−3^). These results advance our understanding of the genetic susceptibility to oligoasthenospermia and more functional studies are needed to provide insights into its pathogenic mechanism.

## INTRODUCTION

Infertility is one of the most frequently diagnosed diseases in reproductive health area, and male-related problems account for approximately half of all infertility cases [1–2]. A significant proportion of idiopathic male infertility is accompanied by quantitative and/or qualitative abnormalities [3, 4]. Although several genetic factors of nuclear genome have been reported to be involved in spermatogenic impairment [5, 6], only few genetic variants of mitochondrion genome have been identified to be associated with spermatogenesis and sperm maturation [7].

As mitochondria are the major source of ATP, they play critical roles in spermatogenesis, differentiation and optimal functioning of germ cells [8]. The 16,569 bp circular human mtDNA encodes two tRNAs, 22 rRNAs, and 13 polypeptides, which are necessary for the proper assembly and function of the mitochondrial complexes of oxidative phosphorylation (OXPHOS) [9, 10]. Sequence polymorphisms in the human mtDNA are significantly related with the geographic origin of the indigenous populations. These mtDNA variants form clusters of related mtDNA haplotypes defined as mtDNA haplogroups [11]. Certain mtDNA haplogroups are associated with specific disease phenotypes, such as Alzheimer's disease and Parkinson's disease [12, 13]. Several studies have reported that mutations in the mtDNA resulted in either functionless or malfunctioning proteins, hence affected sperm motility in different population [14]. Hence, in this study, we hypothesize that mitochondrial genetic variants may be associated with idiopathic oligoasthenospermia in Han Chinese men.

Recently, we conducted a two-stage study to systematically elucidate the potential role of mtDNA genetic variants in oligoasthenospermia based on using next-generation sequencing (NGS) in the discovery phase and SNPscan in the follow-up validation phase [15]. Two mtDNA genetic variants, m.16179C>T and m. 12361A>G, were identified to be associated with low sperm count or motility [15].

Here we evaluated promising associations in an extended validation using 670 cases and 793 controls. We focused on the single nucleotide polymorphisms (SNPs) that have *P* values ranging from 0.1-0.2 in the solexa sequencing stage, reported in our previous study [15]. These results will advance our understanding of the susceptibility to oligoasthenospermia in Han Chinese men.

## RESULTS

### Characteristics of the study population

The final population consisted of 1463 Han Chinese subjects, composed of 793 fertile controls and 670 patients. The distributions of selected characteristics among the case and control subjects were presented in Table 1. No significant differences were identified between the case group and the control group regarding all selected variables (including age, smoking, drinking, tea consumption and BMI).

**Table 1:** The distributions of selected variables among cases and control subjects

Variables	Frequency	Control fertility/normozoospermia (n = 793)	Oligoasthenospermia (n = 670)	*P*
^a^Age(years), mean±SD	-	28.76±3.55	28.52±4.20	2.36×10^−1^
^b^Smoking	Ever	404 (50.95%)	342 (51.04)	1.00
	Never	389 (49.05%)	328 (48.96)	
^b^Drinking	Ever	412 (51.95%)	352 (52.54)	8.33×10^−1^
	Never	381 (48.05%)	318 (47.46)	
^b^Tea consumption	Ever	437 (55.11%)	368 (54.93)	9.58×10^−1^
	Never	356 (44.89%)	302 (45.07)	
^a^BMI, mean±SD	-	23.62±3.92	23.70±3.04	6.67×10^−1^

^a^ Independent-samples T-test was used to test for differences in continuous variables such as age and body mass index (BMI) between the cases and controls.

^b^ Two-sided chi-squared test was used to test the differences of categorical variables such as drinking, smoking status and tea consumption between cases and controls.

### Mitochondrial DNA haplogroup distribution in case group and control group

To assess whether some genetic background are predisposing to or protecting against spermatogenic impairment, we first investigated the mtDNA haplogroups distributions among the case and control groups in the population. Twelve main haplogroups, including A, B, C, D, F, G, M*, M7, M8, M9, N* and N9 (Supplementary Figure 1), were detected in our study subjects. The detailed mtDNA haplogroups distributions were shown in Table 2. Compared to the control group, no significant distribution difference was found between these two groups. These results demonstrated that the genetic background (mainly mtDNA haplogroups) would not influence the susceptibility of studied subjects to spermatogenic impairment.

**Table 2:** Distributions of mtDNA haplogroups among the control group and the case group

mtDNA haplogroups	Case infertility/oligoasthenospermia (n = 670)		Control fertility/normozoospermia (n = 793)		OR (95%CI)^a^	*P*^a^
	n	%	n	%		
A	30	4.48%	34	4.29%	1.05 (0.63-1.73)	8.59×10^−1^
B	101	15.07%	104	13.11%	1.18 (0.88-1.58)	2.82×10^−1^
C	25	3.73%	31	3.91%	0.95 (0.56-1.63)	8.60×10^−1^
D	167	24.93%	188	23.71%	1.07 (0.84-1.36)	5.88×10^−1^
F	88	13.13%	115	14.50%	0.89 (0.66-1.20)	4.51×10^−1^
G	27	4.03%	33	4.16%	0.97 (0.58-1.63)	8.99×10^−1^
M*	45	6.72%	57	7.19%	0.93 (0.62-1.39)	7.24×10^−1^
M7	47	7.01%	60	7.57%	0.92 (0.62-1.37)	6.87×10^−1^
M8	43	6.42%	42	5.30%	1.23 (0.79-1.90)	3.61×10^−1^
M9	10	1.49%	15	1.89%	0.79 (0.35-1.76)	5.57×10^−1^
N*	20	2.99%	20	2.52%	1.19 (0.63-2.23)	5.88×10^−1^
N9	33	4.93%	37	4.67%	1.06 (0.65-1.71)	8.17×10^−1^
Others	34	5.07%	57	7.19%	0.69 (0.45-1.07)	9.54×10^−2^

^a^ ORs and *P* value were obtained from multivariate logistic regression analysis.

### Validation of oligoasthenospermia susceptible mtDNA variants

Through NGS of mtDNA genome, six SNPs met the selection criteria for the validation stage (Table 3). Additive models of logistic regression analyses were used to estimate the *P* values of association analyses. For the exploratory purpose and due to a relatively small sample size in this analysis, 0.1<P≤0.2 were considered statistically suggestive. The frequency distribution of these six SNPs identified in the whole mitochondrial genome sequencing and the validations were shown in Table 4. The frequency distribution of m.11696G>A was at a significantly increased risk of oligoasthenospermia compared with the controls (OR 2.21, 95%CI 1.21-4.04) (*P* = 7.90×10^−3^). To the other genetic variants, no significant differences of distribution frequencies were identified between the two groups.

**Table 3:** Screening predisposed mtDNA variations through solexa sequencing

Gene	Position	Variant	Amino acid	Case infertility/oligoasthenospermia (233)	Control fertility/normozoospermia (233)	OR (95%CI)^a^	*P*^a^
ND1	3398	T to C	M-T	233/0	230/3	-	1.24×10^−1^
ND2	5263	C to T	A-V	226/7	229/4	2.37 (0.61-9.30)	1.69×10^−1^
CO2	7805	G to A	V-I	230/3	233/0	-	1.24×10^−1^
ATP6	9053	G to A	S-N	210/23	217/16	1.49 (0.76-2.89)	1.58×10^−1^
ATP6	9128	T to C	I-T	229/4	225/8	0.49 (0.15-1.65)	1.91×10^−1^
ND4	11696	G to A	V-I	220/13	227/6	1.12 (0.83-5.99)	1.01×10^−1^

^a^ ORs and *P* value were obtained from multivariate logistic regression analysis.

**Table 4:** Association of six identified genetic variations in solexa sequence with oligoasthenospermia according to sperm concentration

Position	Genotype	Control	Case/idiopathic infertility								
			Oligoasthenospermia			Sperm concentration					
						≥15×10^6^/ml			<15×10^6^/ml		
			n (670)	OR (95%CI)^a^	*P*^b^	n (287)	OR (95%CI)^a^	*P*^b^	n (383)	OR (95%CI)^a^	*P*^b^
3398	T/C	783/10	666/4	0.47 (0.15-1.51)	2.82×10^−1^	285/2	0.55 (0.12-2.52)	7.43×10^−1^	381/2	0.41 (0.09-1.89)	3.56×10^−1^
5263	C/T	784/9	660/10	1.33 (0.54-3.29)	5.38×10^−1^	285/2	0.61 (0.13-2.85)	7.37×10^−1^	375/8	1.86 (0.71-4.85)	2.02×10^−1^
7805	G/A	792/1	666/4	4.76 (0.53-42.66)	1.85×10^−1^	286/1	2.77 (0.17-44.42)-	4.61×10^−1^	380/3	6.25 (0.65-60.31)	1.04×10^−1^
9053	G/A	742/51	615/55	1.30 (0.88-1.93)	1.91×10^−1^	264/23	1.27 (0.76-2.11)	3.63×10^−1^	351/32	1.33 (0.84-2.10)	2.27×10^−1^
9128	T/C	781/12	663/7	0.69 (0.27-1.76)	4.30×10^−1^	284/3	0.69 (0.19-2.45)	7.71×10^−1^	379/4	0.69 (0.22-2.14)	6.02×10^−1^
11696	G/A	776/17	**639/31**	**2.261 (1.21-4.04)**	**7.90×10^−3^**	274/13	2.17 (1.03-4.52)	3.50×10^−2^	365/18	2.25 (1.15-4.42)	1.56×10^−2^

^a^ ORs were obtained from multivariate logistic regression analysis.

^b^ P value was obtained from multivariate logistic regression analysis. The significance level was set to 0.05/6 = 0.008, using a Bonferroni correction.

To uncover the exact role of the genetic variants in spermatogenesis, two main sperm parameters (sperm count and motility) were taken into consideration. According to these two parameters, the cases were further classified into two subgroups respectively. As shown in Table 4, the frequency of m.11696G>A was higher in the case group (with sperm concentration <15×10^6^/ml) than that in the control group (OR 2.25; 95 % CI 1.15-4.42) (*P* = 1.56×10^−2^), although the significant differences were not retained after Bonferroni adjustment. As to the sperm motility, logistic regression analysis revealed that only m.11696G>A was associated with a significantly increased risk of asthenospermia (characterized by reduced sperm motility, with sperm motility <40% motile sperm) [16] with the OR value 2.38 (95% CI 1.27-4.46) (*P* = 5.22×10^−3^) (Table 5). Due to the low occur frequency and limited sample size in this study, although m.3398C>T showed decreased risk of asthenospermia (*P* = 8.40×10^−3^), validations are still needed in a larger population (Table 5).

**Table 5:** Association of six identified genetic variations in solexa sequence with oligoasthenospermia according to sperm motility

Position	Genotype	Control	Case/idiopathic infertility								
			Oligoasthenospermia			Sperm motility					
						≥40^a^			<40^a^		
			n (670)	OR (95%CI)^a^	*P* ^b^	n (166)	OR (95%CI)^a^	*P*^b^	n (504)	OR (95%CI)^a^	*P*^b^
3398	T/C	783/10	666/4	0.47 (0.15-1.51)	2.82×10^−1^	162/4	1.93 (0.60-6.24)	2.94×10^−1^	504/0	-	8.40×10^−3^
5263	C/T	784/9	660/10	1.33 (0.54-3.29)	5.38×10^−1^	164/2	1.06 (0.23-4.96)	1.00	496/8	1.41 (0.54-3.67)	6.18×10^−1^
7805	G/A	792/1	666/4	4.76 (0.53-42.66)	1.85×10^−1^	166/0	-	1.00	500/4	6.33 (0.71-56.85)	7.81×10^−2^
9053	G/A	742/51	615/55	1.30 (0.88-1.93)	1.91×10^−1^	152/14	1.34 (0.72-2.48)	3.95×10^−1^	463/41	1.29 (0.84-1.97)	2.44×10^−1^
9128	T/C	781/12	663/7	0.69 (0.27-1.76)	4.30×10^−1^	166/0	-	2.39×10^−1^	497/7	0.92 (0.36-2.34)	1.00
11696	G/A	776/17	**639/31**	**2.261 (1.21-4.04)**	**7.90×10^−3^**	160/6	1.71 (0.66-4.41)	2.64×10^−1^	**479/25**	**2.38 (1.27-4.46)**	**5.22×10^−3^**

^a^ ORs and *P* value were obtained from multivariate logistic regression analysis.

^b^ P value was obtained from multivariate logistic regression analysis. The significance level was set to 0.05/6 = 0.008, using a Bonferroni correction.

## DISCUSSION

The role of mitochondria in spermatogenesis has been extensively researched [17, 18]. However, former studies paid close attention to single or specific genetic variants of mitochondrial genes participated in spermatogenesis [19, 20]. To systematically explore the role of whole mtDNA genome on spermatogenesis, NGS was applied in 233 idiopathic oligoasthenospermia cases and 233 healthy controls, and susceptible genetic variants were evaluated with SNPscan in 670 cases and 793 controls.

It has been demonstrated that mtDNA haplogroup R was a strong independent predictor of sperm motility [21], while others thought that there was no effect of mtDNA haplotype on sperm velocity [22]. Considering above, the frequency distribution of mtDNA haplogrups between the two groups were firstly investigated to study whether population heterogeneity were confounders in identifying candidate mtDNA genetic variants on spermatogenic impairment. In our study, most of these haplogroups are the subgroups of macro-haplogroup M and macro-haplogroup N. The haplogroup frequencies are in line with the frequencies in East Asia. And no significant differences were found in the mtDNA haplogroups distribution. It might be caused by investigating two different cases, asthenospermia and oligoasthenospermia, respectively. Also, Ruiz-Pesini supported that asthenozoospermia, but not oligozoospermia, was associated with mtDNA haplogroups in whites [23]. In other words, the genetic backgrounds may not affect our results of the present association study.

**Figure 1:**
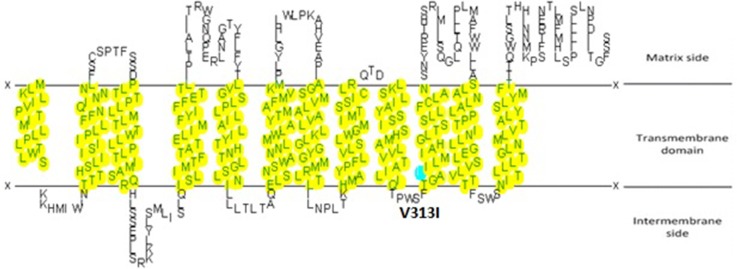
The secondary structure changes of variant m. 11696G>A are predicted by the SOSUI system (http://sosui.proteome.bio.tuat.ac.jp) m.11696G>A would replace the amino acid residue valine at position 313 with isoleucine, which lies in the transmembrane region of the *MT-ND4* subunit.

**Figure 2:**
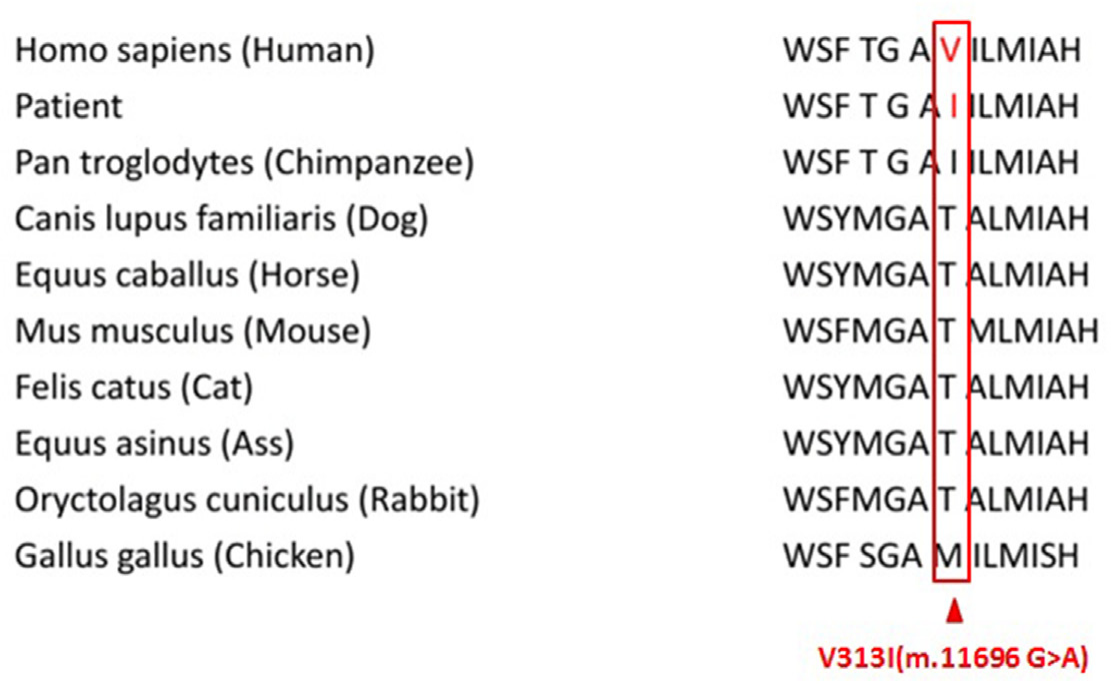
Alignment of the ND4 protein in different species showing the conservation of the amino acid 313

Our results demonstrated that genetic variant m.11696G>A was associated with risk of asthenospermia. The G-to-A transition at position 11696 (m.11696G>A) in the *MT-ND4* gene resulting in the substitution of an isoleucine for valine at amino acid position 313 is located in the predicted transmembrane region (Figure 1) [24]. Besides, it was reported that this mutation was related to mitochondrial diseases such as Leber hereditary optic neuropathy with dystonia and deafness [25–27]. This LHON-associated mtDNA mutation was first identified to be heteroplasmy in a large Dutch family [24]. Interspecies comparison does not show significant conservation of this valine, and in most mammals a threonine residue is found at this position (Figure 2).

Complex I (NADH-ubiquinone oxidoreductase) is the major entry point of electrons into the electron transport chain and contributes to the establishment of a proton gradient that is required for the bulk of cellular ATP synthesis [28]. This enzyme, has an overall L-shaped structure, with one arm, which contains all the subunits encoded by the mtDNA, buried in the mitochondrial inner membrane, and the other arm, which contains the catalytic center, protruding into the mitochondrial matrix [29]. Complex I is composed of 45 subunits, including mitochondrial encoded NADH dehydrogenase subunit (MTND) genes [30]. The seven MTND genes (*MTND1, MTND2, MTND3, MT-ND4L, MT-ND4, MTND5, MTND6*) comprise 38% of the total mtDNA, spanning over 6000 bases of the mitochondrial genome [31]. Complex I accepts electrons from NADH, transfers them to ubiquinone (coenzyme Q10) and uses the energy released to pump protons out across the mitochondrial inner membrane [32]. Defects of complex I are the most common biochemical abnormalities in patients with mitochondrial respiratory chain disorders. The first-ever mtDNA point mutation was described in an MTND gene, and over the past 17 years, lots of pathogenical point mutations in the *MT-ND4* gene have been reported [33–35].

In brief, our study demonstrated that genetic variant m.11696G>A increased the risk of asthenospermia. It is reasonable to surmise that some mtDNA genetic variants in the NADH dehydrogenase genes may cause spermatogenesis failure by decreasing activities of mitochondrial respiratory chain complexes. These findings may contribute to understanding the etiology of male infertility and further functional studies are still needed to support our findings.

## MATERIALS AND METHODS

### Study population and sample collection

This study was approved by the Ethics Review Board of Nanjing Medical University. The protocol and consent form were approved by the Institutional Review Board of Nanjing Medical University prior to the study. After the study procedures were explained and all questions were answered, all of the subjects signed informed consent forms.

We performed a two-stage case-control analysis. The whole mitochondrial genome solexa sequencing phase included 233 idiopathic oligoasthenospermia cases and 233 healthy controls which were consecutively recruited from Affiliated Hospitals of Nanjing Medical University (NJMU Infertile Study). The details of solexa sequencing were described in Lu et al 2015. For validations, we enlarged the sample size by testing 670 oligozoospermia patients and 793 controls, which were continuously enlisted from Renji Hospital. All infertile male subjects were genetically unrelated Han Chinese men and selected based on an andrological examination, including examination of medical history, physical examination, semen analysis, scrotal ultrasound, hormone analysis, karyotyping and Y-chromosome microdeletion screening. Those with a history of cryptorchidism, vascular trauma, orchitis, obstruction of the vas deferens, abnormalities in chromosome number or microdeletions of the azoospermia factor region on the Y chromosome were excluded from the study [36]. All controls, which had a normal reproductive history and normal physical examination, had children within 1 year. After completing a questionnaire, each subject donated 5 ml of blood as a source of genomic DNA for further genotyping analysis.

### Semen analysis

Semen analysis for sperm concentration and motility was conducted on the basis of the World Health Organization (WHO) criteria [37]. To ensure the reliability of diagnosis, each subject was examined twice. The semen parameters (sperm concentration and sperm motility) were dichotomized based on WHO reference values. The controls consisted of proven fertile men with normal semen parameters. Considering the effect of mtDNA genetic variant on sperm concentration or sperm motility separately, we stratified the case group into two sub-groups respectively: concentration-group I (with sperm concentration ≥ 15×10^6^/ml) and concentration-group II (with sperm concentration < 15×10^6^/ml), motility-group I (sperm motility ≥ 40% motile sperm) and motility-group II (sperm motility < 40% motile sperm) [37].

### SNP selection and genotyping for validation

Through NGS of mtDNA genome, variations in each person were scored relative to the revised Cambridge reference sequence (rCRS) [38], and individual haplogroup was defined according to the reported East Asian mtDNA phylogenetic tree [39]. We selected SNPs from the same analysis as Lu et al 2015, meeting the following criteria for the validation: (i) SNPs had 0.1<P≤0.2 in the comparison between 233 cases and 233 controls; (ii) SNPs were potentially functional.

Selected genetic variants were genotyped by a custom-by-design 48-Plex SNPscan^TM^ Kit (Cat#:G0104; Genesky Biotechnologies Inc., Shanghai, China). This kit was developed according to patented SNP genotyping technology by Genesky Biotechnologies Inc., which was based on double ligation and multiplex fluorescence PCR [40]. In order to validate the genotyping accuracy using SNPscan^TM^ Kit, five percent of the samples were randomly selected for repeat genotyping by single nucleotide extension using the Multiplex SNaPshot Kit (Applied Biosystems Inc., Foster City, CA, USA), and the concordance rates were more than 99%.

### Statistical analysis

Basic descriptive diversity statistics were calculated with DnaSP. The association analysis of stage one was performed using PLINK (version 1.07; http://pngu.mgh.harvard.edu/∼purcell/plink/). Statistical analysis of stage two was performed by Stata 10.0 (StataCorp LP, USA). Infertility risks were estimated with odds ratios (OR) and 95% confidence intervals (95% CI) using multivariate logistic regression. Two-sided tests were utilized and the Bonferroni adjustment for multiple testing was used. Thus if there are n tests in a particular set of interest and an investigation-wide 5% test is required, the applied *P* value for a truly significant result is calculated as 0.05/n. Results will also be commented for which, although not attaining significance after applying the rather stringent Bonferroni adjustment, nevertheless returned a spot *P* value of < 5%.

## SUPPLEMENTARY MATERIALS FIGURES AND TABLES


