# Supplementary material for: Mitochondrial DNA sequencing and large-scale genotyping identifies MT-ND4 gene mutation m.11696G>A associated with idiopathic oligoasthenospermia
Source: Oncotarget. 2017 May 8;8(32):52975–82. doi: 10.18632/oncotarget.17675 (PMC5581086; doi:10.18632/oncotarget.17675)
Supplement: Supplementary file 1 [file oncotarget-08-52975-s001.pdf]

Mitochondrial DNA sequencing and large-scale genotyping identifies *MT-ND4* gene mutation m.11696G>A associated with idiopathic oligoasthenospermia

SUPPLEMENTARY FIGURE

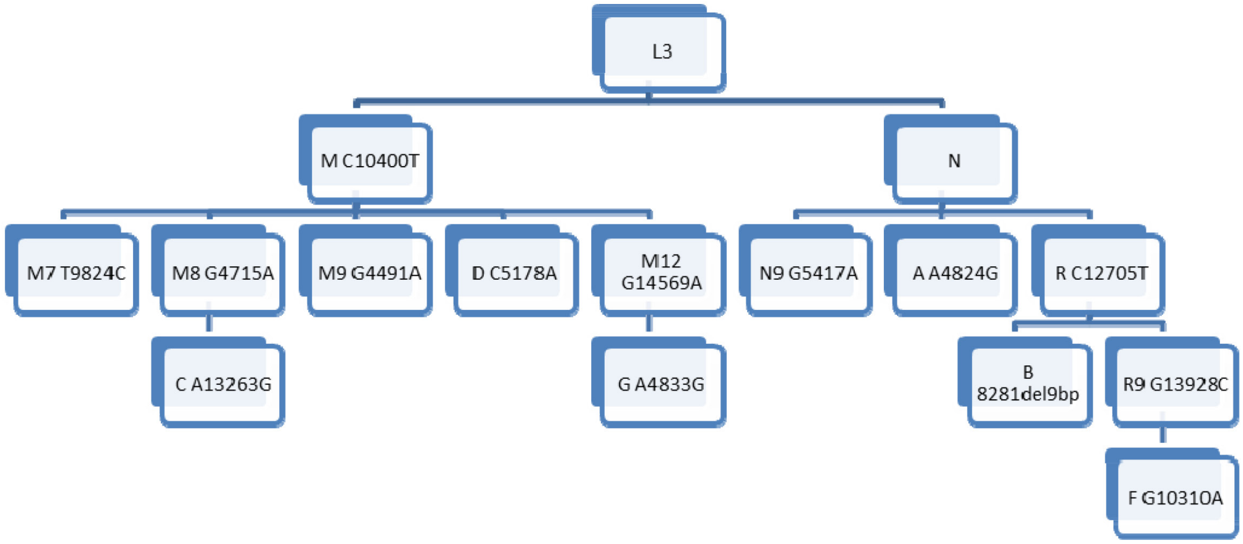

Supplementary Figure 1: The phylogenetic tree of mtDNA haplogroups in the study population. Based on the PhyloTree (http://www.phylotree.org; mtDNA tree Build 16, 19 Feb 2014) and the major East Asia haplogroups.
